# Formulation of Menthol-Loaded Nanostructured Lipid Carriers to Enhance Its Antimicrobial Activity for Food Preservation

**DOI:** 10.15171/apb.2017.031

**Published:** 2017-06-30

**Authors:** Parizad Piran, Hossein Samadi Kafil, Saeed Ghanbarzadeh, Rezvan Safdari, Hamed Hamishehkar

**Affiliations:** ^1^Biotechnology Research Center, Tabriz University of Medical Sciences, Tabriz, Iran.; ^2^Infectious and Tropical Diseases Research Center, Tabriz University of Medical Sciences, Tabriz, Iran.; ^3^Drug Applied Research Center, Tabriz University of Medical Sciences, Tabriz, Iran.; ^4^Zanjan Pharmaceutical Nanotechnology Research Center, and Department of Pharmaceutics, Faculty of Pharmacy, Zanjan University of Medical Sciences, Zanjan, Iran.

**Keywords:** Menthol, Nanostructure lipid carriers, NLC, Antimicrobial activity, Food preservative

## Abstract

***Purpose:*** Due to the antimicrobial property, menthol have significant potential for food preservation and foodstuff shelf life improvement. Nevertheless, menthol instability, insolubility, and rapid crystallization in aqueous media make it unsuitable for used in food products. This work was aimed to prepare menthol-loaded nanostructured lipid carriers (NLCs) to enhance its antimicrobial activity.

***Methods:*** Morphology, particle size and size distribution, encapsulation efficiency percent (EE%), and physical stability of the optimized formulation, prepared by hot melt homogenization method, were characterized by scanning electron microscopy, particle size analyzing, gas chromatography, and X-ray diffraction (XRD) methods. Minimum inhibitory concentration and minimum bactericidal concentration of menthol-loaded NLCs were evaluated and compared with conventional menthol emulsion against various Gram-positive (*Staphylococcus aureus, Bacillus cereus*) and Gram-negative bacteria (*Escherichia coli*), as well as one fungus (*Candida albicans*).

***Results:*** Menthol-loaded NLCs were spherically shaped nanosized (115.6 nm) particles with narrow size distribution (PDI = 0.2), suitable menthol EE% (98.73%), and appropriate physical stability after 90 days of storage period. XRD results indicated that menthol was in the amorphous form in the nanoparticles matrix. Antibacterial assay results revealed that the menthol-loaded NLCs exhibited significantly higher *in vitro* antimicrobial property than conventional menthol emulsion. The results also indicated that menthol-loaded NLCs had better effect on fungi than bacteria, and furthermore, antibacterial efficiency on Gram-positive bacteria was higher than Gram-negative bacteria.

***Conclusion:*** In conclusion, NLCs could be a promising carrier for improvement of antimicrobial activity and preservation efficacy of essential oils in foodstuffs.

## Introduction

Increased concern of consumers on the various side effects of synthetic preservatives and development of antibiotic resistant strains have attracted great attention to the use of natural compounds with antimicrobial activity in food and pharmaceutical industries.^1^ Essential oils are volatile, natural, aromatic oily liquids that can be obtained from several parts of the plants such as leaves and flowers. Due to the aromatic property of essential oils, they have been widely used in cosmetic industries for the production of soaps, perfumes, and toiletries.^2^ The large bioactivity of essential oils has been confirmed by several studies, including antibacterial, antiviral, anti-inflammatory, antifungal, antimutagenic, anticarcinogenic, and antioxidant activities. This group of oils may provide the natural antimicrobials that food industry requires leading to reduced need for synthetic preservative excipients.^3,4^ The main constituents of essential oils have unsaturated carbon chains, which is well-known that are susceptibility to oxidation mediated by light or heat. The high volatility of essential oils also limits their free use without a vehicle. Furthermore, the low aqueous solubility limits the possible application of essential oils in aqueous based foodstuffs such as beverages. All of these factors limit the application of essential oils as candidates for preserving food solutions.^5^ Menthol‏ is a monocyclic monoterpene alcohol naturally obtained from peppermint or other mint oils. It is widely used as a flavoring agent for toothpaste, hygiene products, chewing gum, etc. Menthol is generally available in the form of crystals or granules with a melting point at 41-43 °C. Although, previous researches have shown that menthol has antibacterial and antifungal activity, its high volatility, instability, insolubility, and rapid crystallization in aqueous mediums are the crucial problems concerning its applications and shelf life.^6^ The microencapsulation method is an appropriate technique to solve above mentioned problems. In this perspective, encapsulation procedures provide an effective protection of naturally compounds against chemical reactions and undesirable interactions with other components in food. Furthermore, it improves solubility, diminish migration, and preserve the bioactive compounds stability during food processing and storage.^7^ In food engineering, protection of bioactive compounds such as vitamins, antioxidants, proteins, and lipids could be achieved using nano encapsulation technique to produce functional foods with enhanced functionality and stability.^8^ As lipid based nanoparticles are composed by lipids, they have the ability of interaction with several bacterial and fungal cell types.^9,10^ Therefore, in the present study menthol-loaded nanostructured lipid carriers (NLCs) were prepared to improve the solubility, stability, and antimicrobial efficacy of menthol for potential application as preservative in food industry.

## Materials and Methods

### Materials

Menthol, Tween^®^ 80, Mueller Hinton Agar, Mueller Hinton Broth and Nutrient dextrose agar were supplied from Merck Chemicals Co. (Germany). Poloxamer^®^ 407 was obtained from Sigma-Aldrich Company (USA). Glyceryl Palmtostearate (Precirol ATO-5^®^) was kindly donated from Gattefossé Company (France). Miglyol^®^ 812 was prepared from Sasol Company (Germany). All other used chemicals and reagents were of analytical grade.

#### Preparation of menthol-loaded NLCs and emulsion

Menthol-loaded NLCs were prepared by hot melt homogenization method as described previously.^11^ Briefly, 200 mg menthol was dissolved in 200 mg Miglyol^®^ and added to 1.8 g melted Precirol^®^at about 70 °C. Subsequently, Poloxamer^®^ (1.5 g) was dissolved in water and added drop-wise into the oil phase under stirring at 20000 rpm by a homogenizer (DIAX 900, Heidolph, Germany). Finally, after 15 min, keeping the temperature at 70 °C and the same stirring rate allowed the hot formulation to cool down at room temperature. NLC structures are usually composed of solid lipid (e.g. Precirol^®^) and liquid lipid (e.g. Miglyol^®^). To have an equivalent comparison between NLC and emulsion in their antimicrobial activity evaluation, a similar liquid lipid was chosen for the preparation of NLC and emulsion. Emulsion was also prepared by gradual water addition into the mixture of menthol (200 mg), Miglyol^®^ (2 g), and Tween^®^ 80 (200 mg) under stirring with homogenizer (5000 rpm). Each formulation was prepared and characterized in triplicate.

#### Characterization of menthol-loaded NLCs

Particle size distribution of prepared NLCs was analyzed using dynamic light scattering system (DLS) and reported as intensity-weighted average (z average) and polydispersity index (PDI). Zeta potential of prepared nanoparticles was also analyzed by the same system (Nano ZS, Malvern, UK). The morphology of prepared nanoparticles was obtained using scanning electron microscope (SEM) (LEO 1430VP, UK & Germany) operating at 15 kV. The specimens were mounted on a metal stub with double-sided adhesive tape and coated under vacuum with gold (100-150 A°) in an argon atmosphere prior to observation using a direct current sputtering technique (DST3, Nanostructured Coating Co., Tehran, Iran. In order to assess the effect of nanoparticles’ preparation process on crystallographic patterns of menthol and lipids, XRD analysis was performed using an X-ray diffractometer (D-5000, Siemen, Germany, 2° to 40°) at a scan rate of 0.05 °/s to assess the crystalline structures of menthol, Precirol^®^, and Poloxamer^®^ and optimized NLCs formulation.

#### Determination of encapsulation efficiency (EE%) and loading capacity (LC%) 

The EE% and LE% values were expressed as the percentage of encapsulated menthol to the added menthol or to the used lipid, respectively. EE% was determined by the first separation of the un-entrapped menthol by centrifugation method using Amicon^®^ Ultra-15 with MWCO of 100 kDa (Millipore, Germany) tube. The formulation was added to the upper chamber of the Amicon^®^ tube, and then the tube was centrifuged (Sigma 3K30, Germany) at 5000 rpm for 5 minutes. The clear solution in the bottom of Amicon^®^ tube was used for the determination of unloaded menthol. The EE% and LC% of menthol-loaded NLCs formulations were calculated according to the following equations:

(1) $$EE\left(\%\right)=\frac{{\text{W}}_{\left(Initial\;Menthol\right)}-{\text{W}}_{\left(Free\;Menthol\right)}}{{\text{W}}_{\left(Initial\;Menthol\right)}}\times 100$$

(2) $$\text{LC}\left(\%\right)=\frac{{\text{W}}_{\left(\text{Entrapped Menthol}\right)}}{{\text{W}}_{\left(Total\;lipid\right)}}\times 100$$

where, in equation (1), W_(Initial Menthol)_ is the amount of initial menthol used, and W_(Free Menthol)_ is the amount of free menthol detected in the lower chamber of Amicon^®^ tube after centrifugation of the NLCs formulation (extracted by ether in triplicate). Accordingly, in equation (2), W _(Entrapped Menthol)_ is the amount of loaded menthol, and W_ (Total lipid)_ is the amount of used lipid in the preparation process. The rinsed formulation was used for the further experiments.

#### Physical stability

Samples (15 mL) of each formulation were stored in plastic tubes in the dark place at room temperature (25 ± 1 °C), and the physical stability of the menthol-loaded NLCs was evaluated in terms of the mean particle size of NLCs after 90 days.

#### Gas chromatography

The concentration of menthol was determined using gas chromatography method (Fisons 8160, Milan, Italy, equipped with a flame ionization detector). The DB5 capillary column (30 m in length, 0.25 mm diameter, and 0.25 μm film thickness) was used to separate and quantify the sample compounds. The carrier gas (Helium 99.999%) flow rate was 2.0 mL/min. The temperatures were as follows: injector = 250 °C, detector = 250 °C, column = 50-250 °C (4 °C/min) and injected volume = 1 μL. Data were collected and processed by a computer equipped with Chromcard software (Fisons, Milan, Italy). The reference standard of the menthol was accurately weighed and dissolved in diethyl ether to produce the stock standard solution and was subsequently diluted to a series of appropriate concentration for construction of calibration curves and determination of the limit of quantification (LOQ). The internal standard solution was added in each concentration solution. Calibration curves were constructed with six different concentrations by plotting the peak area ration of analyte to internal standard versus analyte concentration. Calibration curves showed good linearity (0.9966) over a relatively wide concentration range 3.0-7.5 mg/100 mL, and LOQ was calculated as 0.134 mg/mL.

#### Microbial strains

The antimicrobial activity of the menthol essential oil formulations was tested against four food-related microorganisms including one Gram-negative bacterium (*Escherichia coli* ATCC 25922), two Gram-positive bacteria (*Staphylococcus aureus* ATCC 25923 and *Bacillus cereus* ATCC 11778), and fungi (*Candida albicans* ATCC 10231). All the microorganisms were provided by the microbiological laboratory, Drug Applied Research Center, Tabriz University of Medical Science, Tabriz, Iran. After activating, the cultures of bacteria were maintained in their appropriate agar media at 4 °C and used as stock cultures. A single colony from the stock plate was transferred into Mueller Hinton Broth and incubated over night at 37 °C. After incubation, the cells were harvested by centrifugation at 3000 rpm for 15 min, washed twice, and re-suspended in saline solution to provide an optical density equal to 0.5 McFarland standard turbidity (equivalent to 10^7^ colony forming units (CFU) /mL of bacterial and 10^8^ CFU/mL of fungi).

#### Determination of minimum inhibitory concentration (MIC) and minimum bactericidal concentration (MBC) of menthol 

The MIC values were assessed using the broth microdilution method by sterile 96-well microtitre plates.^12^ Bacterial strains were cultured overnight at 37 °C in Muller Hinton Broth. Two fold serial dilutions of the menthol-loaded NLCs with medium were prepared in concentration of 4000 to 7.81 µg/mL for assessment of MIC for *E. coli*, *S. aureus*, and *B. Cereus*. 180 μL of prepared diluted solutions was transferred into 96-well microtitre plates, and then 20 µl of standardized microorganism suspensions was added and incubated at 37 °C for 24 h. After incubation time, turbidity of tubes was evaluated to determine bacterial growth, and last dilution with no turbidity (lack of growth) was considered as MIC. There were different control groups: a) media, b) media + menthol free NLC and emulsion, c) media + menthol-loaded NLC and emulsion (without addition of bacteria), d) media + menthol free NLC and emulsion with addition of bacteria, and e) media + bacteria. Turbidity of wells was measured spectrophotometrically (Ultrospec 2000, Pharmacia Biotech, UK) at the wave length of 620 nm. Subsequently, to determine the MBC, samples (5 μL) from tubes in which no growth was observed were cultured in plate (containing Mueller Hinton agar medium) and incubated for 24 h at 37 °C. To determine the MIC value for *C. albicans*, 16 dilution series from 2496 to 4.87 µg/mL of menthol were prepared, and 50 μL of fungi suspension was added and incubated at 37 °C for 24 h, and the MIC value was determined as the lowest concentration of essential oil inhibiting visible growth of fungi on the agar plate when there was visible growth on the control plates.^13^ Each experiment was performed in triplicate. All procedures were performed under sterile conditions. In each test, microorganism strain in Muller Hinton broth (with blank NLCs or emulsion) and Muller Hinton Broth alone were used as positive and negative growth controls, respectively.

#### Statistical analysis

All experiments were repeated in triplicate, and the data were expressed as a mean value ± standard deviation (SD). Statistical analysis was performed using one-way analysis of variance (ANOVA) with multiple comparisons between deposition data using a Tukey honest signiﬁcant difference (HSD) test using SPSS software (version 13.0, Chicago, IL, USA). A P-value <0.05 was considered statistically signiﬁcant.

## Results and Discussion

### Preparation, characterization and stability of menthol formulations

**Figure 1:**
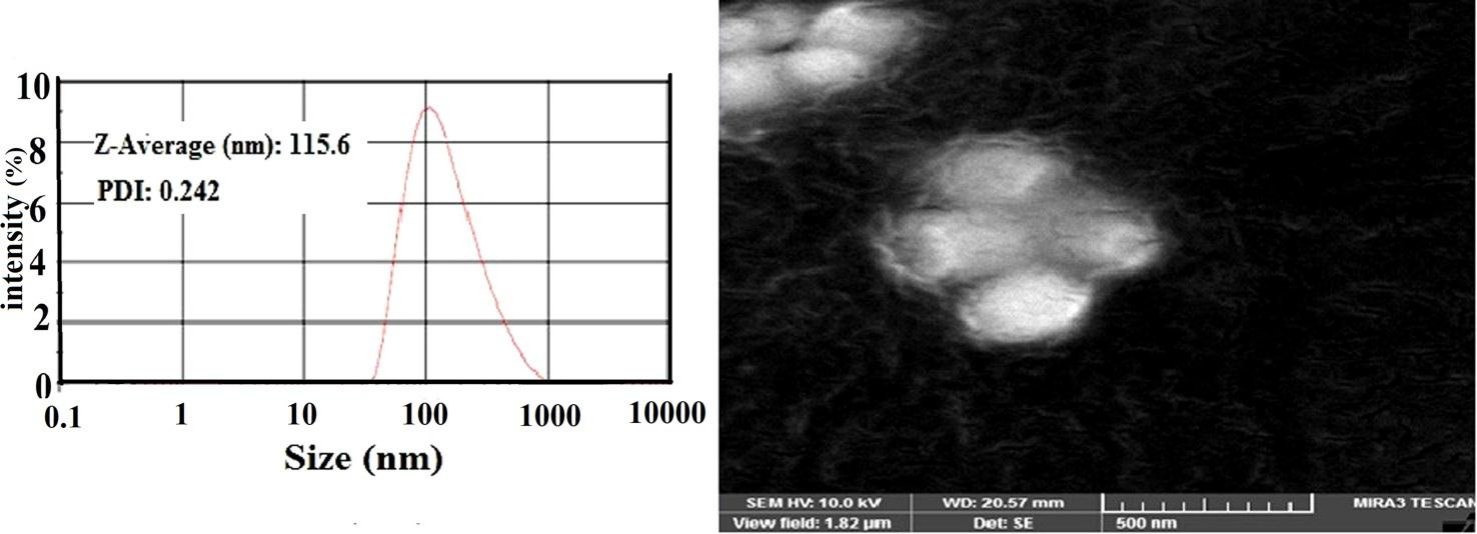
Particle size distribution profile and scanning electron microscopy (SEM) image of optimum nanostructured lipid carriers (NLCs) formulation.

Particle size and size distribution are the key parameters of colloidal systems and have significant effects on the final nanoparticles’ behavior including dissolution, bioavailability, content uniformity, and stability. Dynamic light scattering (DLS) is widely used to determine the size and size distribution of small particles suspended in liquid medium.^14^A PDI value of 0.1 to 0.25 indicates a narrow size distribution whereas a PDI value greater than 0.5 indicates a broad distribution.^15^ Particle size and PDI value of prepared emulsions were 1489 nm and 0.556, respectively. The particle size and size distribution of prepared menthol-loaded NLCs were analyzed, and the size distribution profile of optimal formulation is represented in Figure 1a. The particle size and PDI were in the ranges of 115.6–155.8 nm and 0.243–0.445, respectively. The low PDI value indicates the narrow size distribution of prepared NLCs formulation.^14^ Optimum formulation had z-average value of 115.6 nm and PDI value of 0.243. Lower particle size of nanoparticles resulted in higher clearness of the prepared nano suspension considered as a critical advantage for the‏ drinking stuffs such as beverages. On the other hand, one of the strategies to address stability issues is the particle size; lower particles reduce the sedimentation rate, and therefore, the particles can stay suspended longer in nano suspension formulations. Furthermore, a narrow particle size distribution can minimize the saturation solubility difference and drug concentration gradients within the medium and thus inhibit the occurrence of Ostwald ripening.^16^ SEM images of nanoparticles showed spherical particles which verified the size analysis data of NLCs (Figure 1b). Particle size of menthol-loaded NLCs was found to be affected by the type of surfactant, lipid to surfactant ratio, solid lipid to liquid lipid ratio, and formulation parameters such as temperature and homogenizer speed (data are not shown). Using nanoparticles, the particle size remained as the most important parameter, as many of the chemical and physical properties, such as surface-to-volume ratio inversely proportional to the diameter of the nanoparticles, are strongly dependent on the nanoparticle diameter. Smaller nanoparticles resulted in larger surface area, and therefore, more loading sites were available for applications.^17,18^ Proper concentration of surfactants is also of great impact on the quality of NLCs dispersions. High concentration of emulsiﬁer reduces the surface tension and facilitates the particle partition during homogenization process. In this study, menthol-loaded NLC resulted in the particle size of 136.8 ± 19.1 nm and PDI of 0.23. The PDI value less than 0.3 is considered uniform and narrow size distribution which guarantees reproducible drug delivery. ^15^ The results also indicated that particle size and size distribution of produced nanoparticles were relatively stable during 90 days of storage period (141.8 ± 21.4 and 0.35, respectively), which is a critical advantage for a nanoparticular formulation and guarantees the stability of nanoparticles in distribution mediums such as drinking stuffs.

**Figure 2:**
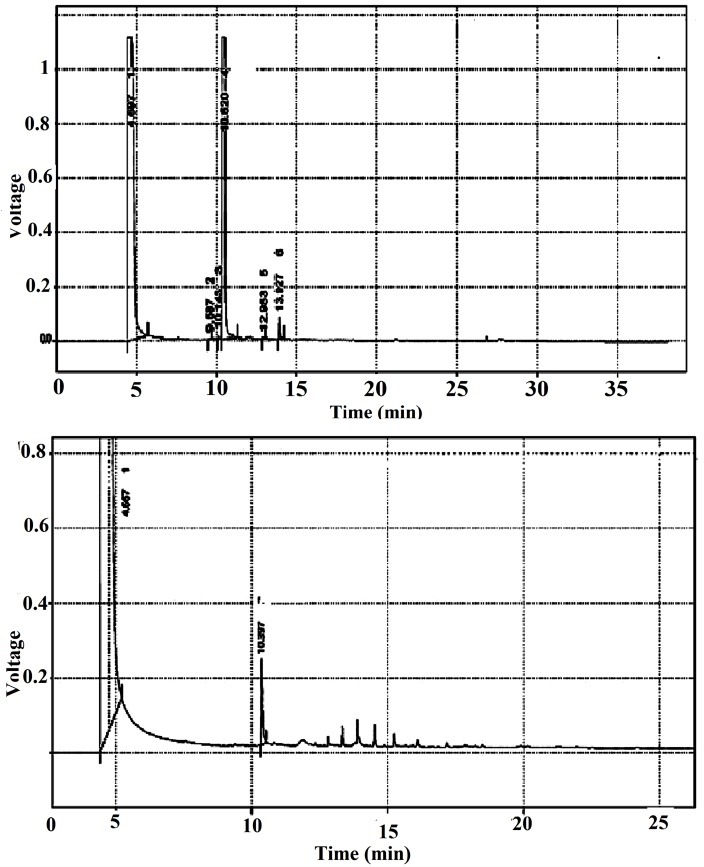
Representative chromatograms of Menthol in Menthol standard solution and Menthol-loaded nanostructured lipid carriers (NLCs).

EE% of optimized NLCs formulation was found 98.73% ± 0.5 with LC% of 9.8% ± 0.05. High EE% was predictable due to the high lipophilicity of menthol and high concentration of Poloxamer^®^ as surfactant in the NLCs formulation. Furthermore, polymorphic and crystallization form of Precirol^®^ in NLCs formulation is another reason for higher EE% value of NLCs.^19^ EE and LC percentage values for emulsion system were achieved 91.4% and 9.1 %, respectively. Representative chromatograms of menthol standard solution and menthol-loaded NLCs formulation after injection into GC column are shown in Figure 2. The peak of menthol was appeared at 10.39 min.

### XRD analysis

**Figure 3:**
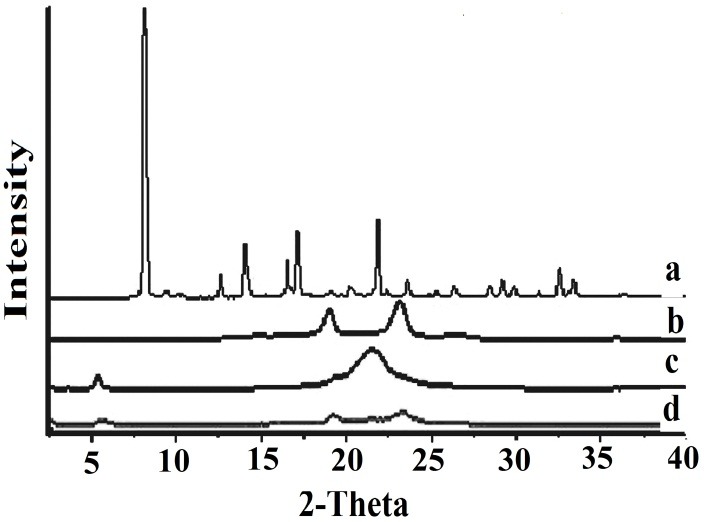
X-Ray diffraction patterns of Menthol, Precirol^®^, Poloxamer^®^ and nanostructured lipid carriers (NLCs) formulation.

The physical state of the nanoparticles was analyzed by X-ray diffraction method (Figure 3). The diffraction pattern of menthol exhibits several sharp peaks, indicating the crystalline nature of menthol. The characteristic main peak for menthol was absent in the XRD pattern of NLCs formulation, suggesting the change from menthol crystallinity status to amorphous status. Diffraction pattern of Precirol^®^ and Poloxamer^®^ in NLCs was much weaker than that of bulk lipid. It indicates that Precirol^®^ in menthol-loaded NLCs was partially recrystallized and formed the less-ordered crystals.

### Determination of the MIC and MBC values of menthol formulations

Plant’s essential oils and extracts have been used for many thousands of years in food preservation, pharmaceuticals, alternative medicine, and natural therapies. It is necessary to scientifically investigate those plants used in traditional medicine to improve the quality of healthcare.^19^ In the present study, the absorbance of microtiter plate before and after the incubation of bacteria, with different concentrations of menthol-loaded NLCs and emulsion, was assessed spectrophotometrically. After incubation and increasing the number of bacteria it is possible to find out the MIC values of menthol-loaded NLCs and emulsion. As shown in Table 1, the growth of Bacillus cereus, Staphylococcus aureus, and Escherichia coli was inhibited at concentration of 125, 250, and 500 µg/mL of menthol in the case of NLCs formulation, respectively. However, the corresponding MIC values for menthol emulsion were 1000, 1000, and 2000 µg/mL, indicating that loading of menthol in NLCs formulation could decrease the amount of necessary menthol for preserving foodstuffs from microorganism growth and spoilage in comparison with emulsion formulation. Addition of low amount of menthol not only is economical, but also may prevent the change in taste and color of the foodstuffs.

**Table 1:** Minimum inhibitory concentration (MIC) and minimum bactericidal concentration (MBC) of Menthol-loaded NLCs and emulsion.

**Microorganism**	**MIC (µg/mL)**		**MBC (µg/mL)**	
	**Emulsion**	**NLCs**	**Emulsion**	**NLCs**
***Staphylococcus *** ***aureus***	1000	125	4000	500
***Bacillus cereus***	2000	250	4000	1000
***Escherichia coli***	2000	500	>4000	>4000
***Candida albicans***	156	78	468	117

**Figure 4:**
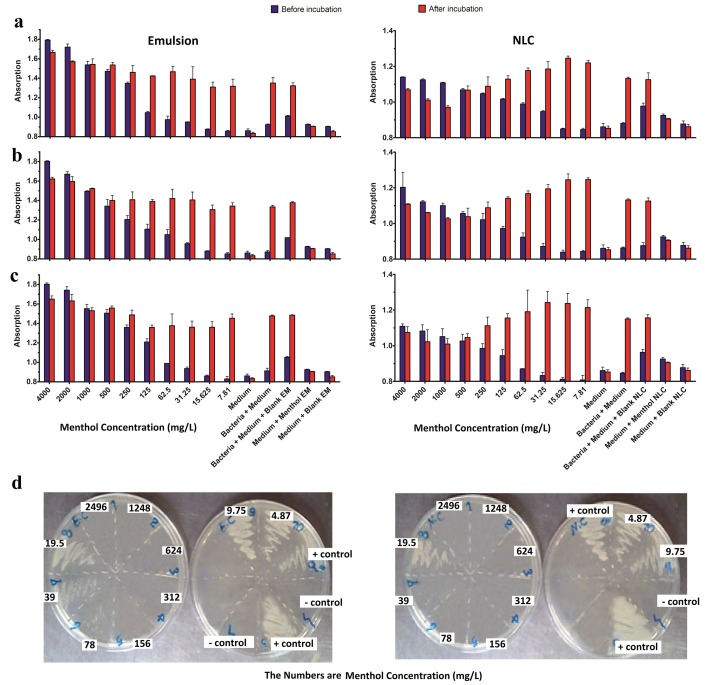
Minimum inhibition concentration (MIC) of conventional emulsion (left) and nanostructured lipid carriers (NLCs) (right) of Menthol against a) *Bacillus cereus, b) Escherichia coli, c) Bacillus cereus and d) Candida albicans*

MIC and MBC values of menthol-loaded NLCs and menthol emulsion formulations against different microorganism are shown in Table 1. Usually the MIC values are considered as different if there is more than one dilution difference between the MIC values. The results showed that in case of the all tested microorganisms when menthol-loaded NLCs were used, the MIC mean values were significantly lower than the when menthol emulsion were used. This suggested the transport mechanisms through the cell membrane of the microorganisms and as a result increasing the antibacterial and antifungal efficiency. On the other hand, menthol-loaded NLCs and emulsion showed lower MIC values, and as a result, higher antibacterial impact on *B. cereus* in comparison with other microorganisms. Correspondingly, both NLCs and emulsion formulations exhibited the highest MIC values in the case of *E. coli*, suggesting that menthol possesses higher antibacterial efficiency against the Gram-positive bacteria (Figure 4).

Antimicrobial effect of menthol may be due to the alteration of plasma membrane permeability and leakage of intracellular materials.^20,21^ However, considering the relation of physicochemical characteristics of the drugs (such as lipophilicity and water solubility), this mechanism appears to be dependent on the lipid composition and net surface charge of the bacterial membranes. Furthermore, the essential oil might cross the cell membranes, penetrate the interior of the cell, and interact with intracellular sites critical for antibacterial activity. The outer layer of the Gram-negative outer membrane is composed primarily of lipopolysaccharide molecules and forms hydrophilic permeability barrier providing protection against the effects of highly hydrophobic materials. This may explain the low sensitivity of Escherichia coli to the cytotoxic effect of the lipophilic monoterpenes such as menthol.^22,23^ NLC formulations also showed promising antifungal activity against candida (Table 1). The successful antifungal activity of NLC loaded with miconazole,^24^ ketoconazole, ^25^ nystatin,^26^ and fluconazole^27^ were also reported indicating the encouraging approach of NLC against candid a. Although several studies showed that menthol and peppermint oil have shown antibacterial activity, the exact mechanism of action is not clearly explained. In a recent study, diamond nanoparticles were used to load menthol against biofilm formation.^28^ The growth of *S. aureus* and *E. coli* made biofilms were inhibited more than 10 folds. The toxic effects on the membrane are often used to reveal the antimicrobial activity of the oil. Trombetta et al. (2005) who explored the mechanism of action of three monoterpenes, speculated that the antimicrobial effect of monoterpenes such as (+)-menthol, thymol and linalyl acetate, is partially due to the disruption of the lipid fraction of the plasma membrane, causing in changed permeability and leakage of intracellular materials.^28,29^ Emulsions are homogeneous thermodynamically stable transparent dispersions of two immiscible liquids stabilized by an interfacial film of surfactants. However, one major drawback of emulsions is that formation requires high surfactant concentration, which causes toxicity when used in pharmaceutical applications. On the other hand, NLCs could be prepared by a simple and industrially scaling up homogenization method. It has been demonstrated that NLCs increase the retention of a drug in the particle, hence, enhancing the release time and reducing the amount of drug required for the therapeutic action. This proficiency could be employed in controlling essential oils release to improve efficacy and reduce the amount and toxicity of used essential oils. Besides, controlling the release of drug molecule, nanocarriers also protect essential oils against possible thermal or photo degradation, oxidation, or evaporation which guarantees increased stability, flavor, and function, consequently prolonging the final product shelf life. Considering these features, these systems can actually represent an interesting approach for overcoming essential oils restrictions. Nanocarriers could also protect essential oils against possible thermal- or photo-degradation, oxidation or evaporation, assure increased stability, flavor, and function, and consequently extend the final product shelf life.^30^

## Conclusion

Menthol was successfully loaded into NLCs in the amorphous structure in the ratio of 1:10 Menthol:lipid by dissolving in the oil phase. Menthol-loaded NLCs were around 100 nm with narrow size distribution (PDI 0.2). The antimicrobial efficiency of the encapsulated menthol was tested on four different microorganisms, demonstrating that MIC and MBC values in the case of NLCs were lower than the menthol emulsion. It can be concluded that essential oils could be used as antibacterial supplement and food preserving agents; however, further investigations are required on the potential antibacterial applicability of this nanostructure in other applications such as topical and oral uses especially against the drug-resistant microorganisms.

## Acknowledgments

This paper was financially supported by Tabriz University of Medical Sciences.

## Ethical Issues

Not applicable.

## Conflict of Interest

The authors declare no conflict of interests.
